# Evaluation of Post Laryngectomy Pharyngocutaneous Fistula risk Factors 

**Published:** 2016-03

**Authors:** Sophia Nitassi, Jihane Belayachi, Mohammed Chihab, Ilham Rkain, Jalila Benayad, Mohammed Anas Benbouzid, Abdelillah Oujilal, Leila Essakalli

**Affiliations:** 1 *Department of Otorhinolaryngology-Head and Neck Surgery, Specialities Hospital, Faculty of Medicine in Rabat; Mohammed V University; Rabat. Morocco.*; 2 *Department of Medical Emergency Care Unit, Ibn Sina Hospital. Faculty of Medicine in Rabat; Mohammed V University; Rabat. Morocco. *

**Keywords:** Incidence, Pharyngeal, Fistula, Risk Factor, Total laryngectomy.

## Abstract

**Introduction:**

Pharyngocutaneous fistula (PCF) is the most common complication after total laryngectomy. Its incidence is extremely variable, with values ranging from 3% and 65%. The management of this problem considerably increases the length and the cost of hospitalization. The aim of this study is to analyze the incidence, predisposing factors, and outcome of PCF in patients undergoing total laryngectomy in a Moroccan teaching hospital in Rabat, Morocco.

**Materials and Methods:**

This study is a retrospective study including 136 patients who underwent total laryngectomy for squamous cell carcinoma of the larynx in our institution, between January 2006 and December 2013. Socio-demographical, biological, surgical, and outcome data were included. Risk factors were analyzed for association with PCF formation.

**Results:**

The overall PCF rate was 27.8%. The mean age was 58 (32-82 years). Univariate analysis showed age (P= 0,028), hemoglobin (P=0,026), and previous tracheotomy (P=0,028) to be associated with the onset of PCF. However, multivariate analysis revealed that previous tracheotomy (P=0,028) and low level of preoperative hemoglobin (P=0,026) were highly associated with the occurrence of PCF.

**Conclusion:**

This is an original work performed in an African country with a large serie. Our findings suggest that age, previous tracheotomy, and low level of haemoglobin are risk factors for PCF onset after total laryngectomy in T4 squamous cell carcinoma.

## Introduction

Total laryngectomy still remains the treatment of choice in T4 laryngeal squamous cell carcinoma (1,2).

The pharyngocutaneous fistulae (PCF) is the most common complication in the postoperative period after total laryngectomy (3). It's an abnormal fistulae track between the pharynx and the cervical skin, which manifests as appearance of saliva on the skin surface after swallowing (4). It occurs typically at the level of a surgical incision, or less frequently around a tracheostoma. It's distinguished from the pharyngostoma, which is a total opening of the pharynx. The continuous flow of saliva is the primary cause of infection that hinders PCF closure. It is a complication that considerably increases the duration and the cost of hospitalization and could delay the starting of the postoperative radiotherapy. It affects the well-being of the patient with a negative psychological impact on quality of life (5).

The etiology of PCF formation is multifactorial and its incidence is extremely variable, with values ranging from 3% and 65% (3). Many investigations have been performed to find out the contributing factors in the development of PCF with the aim of reducing its incidence, but the findings are inconsistent. Several parameters have been considered as predisposing factors to fistula formation in the literature: previous radiotherapy (6,8), types of surgery, combination with neck dissection (6), types of pharyngeal closure and suture material (9), preoperative tracheotomy (10), systemic disease (8), tumor localization (4), low hemoglobin level (7,10), and prophylactic antibiotic use (9). However, some of these are currently under discussion.

The Moroccan health care system includes 122 hospitals, 2,400 health centers, and 4 university clinics, but they are poorly maintained and lack adequate capacity to meet the demand for medical care. Twenty-four thousand beds were available for 6 million patients seeking care each year, including 3 million emergency cases (11). 

Morocco has two major health sectors, public and private, said to be complementary rather than competitive. 

The aim of our study was to determine the incidence of PCF and the risk factors involved in the onset of PCF after a total laryngectomy in our institution.

## Materials and Methods

*Study design*

This was a retrospective study performed in the otorhinolaryngology and head and neck surgery department at Specialties Hospital in Rabat, Morocco from January 2006 to December 2013.

*Settings*

The Specialties Hospital is a teaching hospital, which consists of 4 clinical services: otorhinolaryngology and head and neck surgery, neurology, neurosurgery, ophthalmology with a radiological and biological unit and an administrative service.

The otorhinolaryngology department is the biggest in CHIS. It has the capacity of seventy beds. We practice otology, rhinology, oncology, and maxillofacial surgery. We have hearing and laryngological explorations and daily external consultations of about over than 50 per day.

*Patients*

The study included all patients who underwent an operation in our institution, which consisted of total laryngectomy for squamous cell carcinoma of the larynx in stage T4 regardless of nodal status and if the endolaryngeal space was involved. T4 is defined by a cricoid and/or thyroid cartilage involvement and/or extralaryngeal extension. The study excluded patients undergoing first chemoradiotherapy or salvage laryngectomy and patients with missing data in their medical records. One hundred and thirty-six patients were collected. All of them were men. The mean age was 58±10,65. Approval for this study was obtained from the Rabat University ethics committee.

*Protocol*

All patients received an antibioprophylaxis in the operating room during anesthetic induction, which contained amoxicillin and clavulanic acid to the renewed dose of 1g every 6 hours. It was administered continuously and intravenously for 48 hours at the dose of 1 g / 8h then taken orally for 8 days. The nasogastric tube was maintained for 10 days outside the occurrence of PCF and maintained otherwise until the PCF had healed.

The patients were operated by a senior surgeon who was helped by two junior surgeons. Tree types of pharyngeal closures were performed: extra-mucosal sutures in T4, extra-mucosal vertical closure, or horizontal closure with transmucosal separate points.

An antiemetic was administered during the first 48 hours. Both gastric protection by inhibitors of proton pump and an antiseptic mouthwash for the duration of healing were required 

*Data collection*

Socio-demographic, biological, disease, surgical, and outcome data were collected. Socio-demographic data included age, gender, toxic habits (tobacco, alcohol), and diabetes mellititus. Biological data included blood glucose levels, serum protein levels, and preoperative hemoglobin. Serum protein levels less than 70g/l were considered as hypoprotidemia and hemoglobin level less than 12g/l were considered as anemia. Tumor location (glottic, glotto-susglottic, glotto-subglottic tumor with an extralaryngeal extension), first tracheotomy, type of pharyngeal closure, use of a flap were reviewed. The extralaryngeal extension included soft tissues and/or the pharynx and/or the thyroid gland. The onset of PCF was reported. 

*Data analysis*

Data were analyzed using the SPSS 10.0 software. Continuous variables are presented as mean ± standard deviation for variables with a normal distribution and as median and interquartile range (IRQ) for variables with skewed distributions. The normality of the distribution was tested by the Kolmogorov-Smirnov test with Lillifors correction. For categorical variables, the percentage of patients in each category was calculated.

To demonstrate a statistically significant relationship between the above mentioned variables and the occurrence of PFC, we used Fisher's exact test and Chi- square test for categorical variables and the Student t test for continuous variables with normal distribution. 

Multivariable analyses were performed using logistic regression with varying subsets of predictors based on the univariate analysis. A P-value≤0.05 was considered statistically significant. There is no other similar study done in Morocco.

## Results

*Descriptive analysis*

One hundred thirty-six patients were collected. All of them were men. The mean age was 58±10,65. Six patients had diabetes mellititus. A flap reconstruction was used in 3 cases. It was a major pectoralis flap in all cases. The complete descriptive data was reviewed in (Table.1).

*Analytic analysis*

In the univariate analysis, age, hemoglobin less then 12g/l, and previous tracheotomy were associated with PCF onset. The complete results were reported in (Table. 2).

In the multivariable analysis, after adjustment of hemoglobin levels and age, previous tracheotomy increases PCF onset by two times and half after total laryngectomy for laryngeal T4 squamous cell carcinoma (Table. 3).

**Table1:** descriptive analysis showing sociodemographic, biological, treatment and outcome data

	N=136	
**Age**	58±10,65*	
**Toxic habits**		
tabacco	91	66.9
cannabism	6	4.4
Tabacco+alcohol	35	25.7
none	2	1.5
**Hemoglobin**		
Normal	115	84.6
Abnormal	21	15.4
**Serum protein level**		
>70 g/l	84	58.8
<70g/l	52	38.2
**Blood sugar level**		
Normal	105	77.2
Abnormal	31	22.8
**Tumor location**		
glottic	14	10.3
Glotto subglottic	43	31.6
Glotto sous glottic	29	21.3
Subglottic	5	3.7
3 laryngeal floor	26	19.1
Extralaryngeal extension	17	12.5
**Lymph nodes dissection**		
yes	130	95.6
No	6	4.4
**Use of a flap**		
yes	3	2.2
no	133	97.8
**Pharyngeal closure**		
Horizontal	15	11
T	52	38.2
Vertical	21	15.4
**First tracheotomy**		
yes	72	52.9
No	63	46.3
**PCF**		
**yes**	37	27
**no**	96	73
**PCF duration**		
<4j	3	8.1
>4j	34	91.9

* mean and ecart type

**Table 2:** Univariate Analysis of risk factors in PCF formation

	**pharyngostoma**		**p**
	Yes N(%)	No N(%)	
**Age**	61.29±11.8*	56.68±9.97*	0.025
**Tumor location**			
Glottic	2(5.6)	12(12.6)	
Glottosubglottic	13(36.1)	29(30.5)	
Glottosousglottic	5(13.9)	24(25.3)	NS
Subglottic	1(2.8)	4(4.2)	
Tree floors	8(22.2)	18(18.9)	
Extralaryngeal extension	7(19.4)	8(8.4)	
**Pharyngeal closure**			
horizontal	3(10.3)	11(19.3)	NS
T shaped	18(62.1)	33(57.9)	
vertical	8(27.6)	13(22.8)	
**Flap**			
yes	1(2.7)	2(2.1)	NS
No	36(97.3)	94(97.9)	
**Hemoglobin**			
>12g/l	27(73)	85(85.5)	0.035
<12g/l	10(27)	11(11.5)	
**Serum protein level**			
>70 g/l	21(56.8)	61(63.5)	0.52
<70 g/l	16(43.2)	35(36.5)	
**Blood sugar level**			
normal	26(70.3)	77(80.2)	0.25
Abnormal	11(29.7)	19(19.8)	
**First tracheotomy**			
yes	11(29.7)	49(51.6)	0.032
no	26(70.3)	46(48.4)	

* mean±ecart type

**Table 3:** multivariable analysis of risk factors associated with PCF analytic analysis of our study

	**OR**	**IC 95%**	**P**
Age	1.042	1,000-1.085	0.05
Hemoglobin	4.072	1.414-11,72	0.009
Serum protein level	-	-	NS
Blood sugar level	-	-	NS
First tracheotomy	2.626	1.111-6.207	0.028



OR=odds Ratio; IR=intervalle de confiance

## Discussion

To our knowledge, no data from low- or middle-income countries have been reported and our findings are more likely to be applicable to such countries than findings of studies in developed countries. PCF is the most common complication after total laryngectomy (12). The incidence of PCF is variable, ranged from 5 to 65 % (3). Our incidence was 23.7 % which is in line with the literature data; however, it is still very high compared to data from recent studies done in developed countries which quoted incidence between 10% and 14% (7-8). In our context, this complication increases hospital stay and medical cost, and negatively impacts patients’ quality of life. Simultaneously, it may also impede rehabilitation and delay adjuvant postoperative treatment. This relative high incidence may be explained by the large neck dissection with the bilateral lymph node removed which could lay a large space to fistulae; or maybe a lack in nurse care and patient hygiene after surgery.

Several studies have been performed to find out the contributing factors in the development of PCF with aim of reducing its incidence, but the findings are still conflicting. This can be explained by the different patients’ selections, the sample sizes and methodology employed. 

Our results did not show any significant association between a history of smoking and alcohol consumption and PCF. It was consistent with the previous results (10). It was widely stated that PCF formation was not influenced by age and gender (4). DEDIVITIS et al proposed that fistula development did not correlate with gender but with age and its incidence increased after 60 years (13). Our study showed that advanced age was statistically associated with its development only in univariate analyses. 

Diabetes mellitus was regarded as an important risk factor (6). The consequences of hyperglycemia are glycosuria and impairment of wound healing. These diabetic patients might be at increased risk for adverse outcome. In our study, the high level of blood sugar was not a risk factor for the occurrence of PCF.

There is no consensus on whether tumor location is a significant risk factor for PCF (4,6,13). Some authors found that the incidence of PCF was higher in supraglottic tumors compared to glottic tumors (14). This is because supra-glottic tumors require more extensive resection, including the pharyngeal wall, which may result in more tension on the pharyngeal suture line thus increasing the risk of PCF. Our result did not find any significant association between tumor location and PCF formation.

Our study identified preoperative tracheostomy to be significantly associated with PCF both through univariate analysis and after adjustment for other factors. Several studies had identified previous tracheotomy as a risk factor for PCF occurrence (3-5-10). This is probably due to local inflammation and it also implicitly means that the tumor is advanced (13-15).

In our study, preoperative hemoglobin level (lower than 12g/dl) was associated with the occurrence of PCF. This result is consistent with literature (7). Other factors have been cited in literature such as lymph node status, microscopic invasion of the surgical limits, pharyngeal tumor extension, preoperative radiotherapy, and a duration of operation greater than 10 hours (16,17). 

In the largest case study reported by Betlejewski et al., of about 538 cases, only the general condition of the patient and postoperative antibiotic constituted risk factors of PCF (14). In our study, all patients had the same postoperative antibiotic therapy. 

In the meta analysis of Cecattoa et al. which has taken over than 36 publications on the PCF, the first tracheotomy, hemoglobin less than 12.5g/l, hypoalbumi- nemia , tumor resection margins, radiation therapy, preoperative are risk factors are all reported as risk factors (5). These results are also found in the meta-analysis of Paydarfar et al (6). There is a general consensus that the initial management of fistulae should be conservative, as they close spontaneously in most cases (7,8,10). In our series 95% of patients evolved favourably with local care and tube feeding, a figure which coincides with the published data. Our study had several limitations: first, it was conducted at a single institution; second, it was a retrospective study with missing data; third, we didn't specify the extralaryngeal extension because of the missing data.

## Conclusion

PCF remains a common complication of total laryngectomy. Multiple risk factors have been implicated. However, since the same factors have not appeared to be significant in all studies, controversy still remains in identifying high-risk patients. Our findings suggest that previous tracheotomy and preoperative anemia are risk factors for PCF onset after total laryngectomy in T4 squamous cell carcinoma in our population. Future prospective study will include patients who are undergoing neoadjuvant radiochemi- otherapy to evaluate this factor.

## References

[1] Rosenthal Di, Mohamed As, Weber Rs, Garden As, Sevak Pr, Kies Ms (2015). Long-term outcomes after surgical or nonsurgical initial therapy for patients with T4 squamous cell carcinoma of the larynx: A 3-decade survey. Cancer.

[2] SATOT ( 1985). Treatment of laryngeal cancer Gan No Rinsho.

[3] Mäkitie Aa, Niemensivu R, Hero M, Keski-Säntti H, Bäck L, Kajanti M (2006). Pharyngocutaneous fistula following total laryngectomy: a single institution's 10-year experience. Eur Arch Otorhinolaryngol.

[4] Palomar-Asenjo V, Sarroca Capell E, Tobías Gómez S, Pérez Hernández I, Palomar-García V (2008). Pharyngocutaneous Fistula Following Total Laryngectomy. A Case-Control Study of Risk : Factors Implicated in its Onset. Acta Otorrinolaringol Esp.

[5] Cecattoa Sb, Monteiro Soaresa M, Henriquesa T, Monteirob E, Ferreira Pinto Mourac CI (2014). Predictive factors for the postlaryngectomy pharyngocutaneous fistula development: systematic review. Braz J Otorhinolaryngol.

[6] Paydarfar Ja, Birkmeyer NJ (2006). Complications in head and neck surgery: a meta-analysis of postlaryngectomy pharyngocutaneous fistula. Arch Otolaryngol Head Neck Surg.

[7] Cavalot Andrea L, Gervasio Cf, Nazionale G, Albera R, Bussi M, Staffieri A (2000). Pharyngocutaneous fistula as a complication of total laryngectomy: review of the literature and analysis of case records. Otolaryngol Head Neck Surg.

[8] Saki N, Nikakhlagh S, Kazemi M (2008). Pharyngocutaneous Fistula after Laryngectomy: Incidence,Predisposing Factors, and Outcome. Arch Iranian Med.

[9] Zhang X, Liu Z, Li Q, Liu X, Li H, Liu W (2013). Using a linear stapler for pharyngeal closure in total laryngectomy. Eur Arch Otorhinolaryngol.

[10] Benson Em, Hirata Rm, Thompson Cb, Ha Pk, Fakhry C, Saunders Jr (2015). Pharyngocutaneous fistula after total laryngectomy: A single-institution experience, 2001-2012. Am J Otolaryngol.

[11] World Health Organization: Country Cooperation Strategy for WHO and Morocco 2004-2007.

[12] Timmermans Aj, Lansaat L, Theunissen Ea, Hamming-Vrieze O, Hilgers Fj, Van Den Brekel Mw (2014). Predictive factors for pharyngocutaneous fistulization after  total laryngectomy. Ann Otol Rhinol Laryngol.

[13] Dedivitis Ra, Ribeiro Kc, Castro Ma, Nascimento Pc (2007). Pharyngocutaneous fistula following total laryngectomy. Acta Otorhinolaryngol Ital.

[14] Markou Kd, Vlachtsis Kc, Nikolaou Ac, Petridis Dg, Kouloulas Ai, Daniilidis Ic (2004). Incidence and predisposing factors of pharyngocutaneous fistula formation after total laryngectomy. Is there a relationship with tumor recurrence?. Eur Arch Otorhinolaryngol.

[15] Gonzalez Aguilar O, Pardo Ha, Vannelli A, Simkin Do, Rossi A, Rubino A (2001). Total laryngectomy: pre- and intrasurgical variables of infection risk. Int Surg.

[16] R Schwartz S, Yueh B, Maynard C, Daley J, Henderson W F, Khuri S (2004). Predictors of wound complications after laryngectomy: A study of over 2000 patients. Otolaryngology - Head and Neck Surgery.

[17] Esteban F, Delgado-Rodríguez M, Mochón A, Solano J, Soldado L, Solanellas J (2006). Study of in-patient hospital stay following totallaryngectomy: multivariable retrospective analysis of a 442 total laryngectomies. Acta Otorrinolaringológica Española.

[18] Betlejewski S, Szymańska-Skrzypek (2007). Post-Laryngectomy Pharyngocutaneous Fistula-A Continuing Clinical Problem. A Otolaryngol Pol.

[19] Scotton W, Cobb R, Pang L, Nixon I, Joshi A, Jeannon Jp (2012). Post-operative wound infection in salvage laryngectomy: does antibiotic prophylaxis have an impact?. Eur Arch Otorhinolaryngol.

